# Effects of temperature and humidity on acute myocardial infarction hospitalization in a super-aging society

**DOI:** 10.1038/s41598-021-02369-x

**Published:** 2021-11-24

**Authors:** Takumi Higuma, Kihei Yoneyama, Michikazu Nakai, Toshiki Kaihara, Yoko Sumita, Mika Watanabe, Shunichi Doi, Yoshihiro Miyamoto, Satoshi Yasuda, Yuki Ishibashi, Masaki Izumo, Yasuhiro Tanabe, Tomoo Harada, Hisao Ogawa, Yoshihiro J. Akashi

**Affiliations:** 1 Division of Cardiology, Department of Internal Medicine, St. Marianna University School of Medicine, 2-16-1, Sugao, Miyamae-ku, Kawasaki, Kanagawa 216-8511 Japan; 2 Center for Cerebral and Cardiovascular Disease Information, National Cerebral and Cardiovascular Center, Suita, Osaka Japan; 3 Department of Cardiovascular Medicine, Tohoku University Graduate School of Medicine, Sendai, Miyagi Japan; 4 Kumamoto University, Kumamoto, Japan

**Keywords:** Climate sciences, Cardiology

## Abstract

Weather conditions affect the incidence of acute myocardial infarction (AMI). However, little is known on the association of weather temperature and humidity with AMI hospitalizations in a super-aging society. This study sought to examine this association. We included 87,911 consecutive patients with AMI admitted to Japanese acute-care hospitals between April 1, 2012 and March 31, 2015. The primary outcome was the number of AMI hospitalizations per day. Multilevel mixed-effects linear regression models were used to estimate the association of the average temperature and humidity, 1 day before hospital admission, with AMI hospitalizations, after adjusting for weather, hospital, and patient demographics.Lower temperature and humidity were associated with an increased number of AMI hospitalizations (coefficient − 0.500 [− 0.524 to − 0.474] per °C change, *p* < 0.001 and coefficient − 0.012 [− 0.023 to − 0.001] per % change, *p* = 0.039, respectively). The effects of temperature and humidity on AMI hospitalization did not differ by age and sex (all interaction p > 0.05), but differed by season. However, higher temperatures in spring (coefficient 0.089 [0.025 to 0.152] per °C change, *p* = 0.010) and higher humidity in autumn (coefficient 0.144 [0.121 to 0.166] per % change, *p* < 0.001) were risk factors for AMI hospitalization. Increased average temperatures and humidity, 1 day before hospitalization, are associated with a decreased number of AMI hospitalizations.

## Introduction

Acute myocardial infarction (AMI) is recognized as one of the leading causes of death in the elderly worldwide. Several traditional factors, including age, hypertension, diabetes, hyper cholesterol, and smoking, are associated with the onset of AMI^1^. Furthemore, heavy exercise or physical exertion, diet, sexual activity, emotional stress, and environmental conditions have been associated with AMI^2–4^. Air pollution, influenza epidemics, and weather changes have been reported as environmental triggers of AMI^4–6^. Among the environmental conditions, low temperatures have been reported to be a potentially important risk factor for AMI^4^, and cardiovascular mortality^7,8^. Therefore, meteorological temperature is considered a risk factor for AMI.

Humidity can easily be obtained together with temperature. However, the association between humidity and the risk of AMI is still unclear. This is due to the lack of consensus on the relationship between high and low humidity and the risk of AMI^4,9–12^. In recent years, Japan has become a super-aging society with average life expectancies of 81 and 87 years for males and females, respectively. Consequently, 23.8% of the population die from heart and cerebrovascular diseases (https://www.mhlw.go.jp/english/database/db-hh/1-2.html). However, little is known on the association of weather temperature and humidity with AMI hospitalizations in the super-aging society of Japan.

To address these issues, we conducted an observational study using the Japanese registry of all cardiac and vascular diseases (JROAD) database. The JROAD database is the largest database in Japan in the field of cardiovascular diseases. It contains data for patients who require hospitalization for cardiovascular diseases. We hypothesized that temperature and humidity are associated with AMI hospitalizations, and we expected their effect to vary by age, sex, and season. Until now, there has been a few nationwide research data on the effect of temperature and humidity on the risk of AMI. The present study provides important insights on the risk of AMI in the aging Japanese society.

## Results

### Patient baseline characteristics

The baseline characteristics of the study population are shown in Table 1. The median age was 71.0 (61.0–80.0) years, and 27.8% were female. The median average temperature and humidity 1 day before hospital admission for AMI were 15.0 °C and 71.0%, respectively.

**Table 1:** Baseline characteristics.

N	AMI patients	Patients not included	P-value
	n = 87,911	n = 742,588	
Age, year, median (IQR)	71.0 (61.0, 80.0)	77.0 (66.0, 84.0)	< 0.001
Age ≤ 64, n (%)	29,387 (33.4)	169,712 (22.9)	< 0.001
Age 65–74, n (%)	24,116 (27.4)	153,860 (20.7)	
Age 75–89, n (%)	30,384 (34.6)	345,328 (46.5)	
Age ≥ 90, n (%)	4024 (4.6)	73,688 (9.9)	
Gender, male, n (%)	24,481 (27.8)	325,402 (43.8)	< 0.001
Height, cm, median (IQR)	160.0 (150.0, 168.0)	154.0 (142.0, 163.0)	< 0.001
Weight, kg, median (IQR)	60.0 (49.0, 69.1)	51.0 (39.3, 61.8)	< 0.001
Charlson score, median (IQR)	2.0 (1.0, 3.0)	1.0 (1.0, 2.0)	< 0.001
AMI, n (%)	87,911 (100.0)	0 (0.0)	< 0.001
Angina, n (%)	2235 (2.5)	84,209 (11.3)	< 0.001
UAP, n (%)	0 (0)	45,147 (6.1)	< 0.001
Atrial fibrillation, n (%)	141 (0.2)	26,040 (3.5)	< 0.001
Heart failure, n (%)	2054 (2.3)	242,226 (32.6)	< 0.001
Aortic disease, n (%)	162 (0.2)	45,914 (6.2)	< 0.001
Cardiac arrest, n (%)	2495 (2.8)	70,581 (9.5)	< 0.001
Pulmonary embolism, n (%)	34 (< 1)	9954 (1.3)	< 0.001
Hospital bed count, median (IQR)	467 (341, 621)	464 (330, 631)	0.088
Average temperature 1 day before admission, °C, median (IQR)	15.0 (6.9, 22.3)	14.9 (6.9, 22.3)	0.360
Average humidity 1 day before admission, %, median (IQR)	70.5 (60.0, 80.5)	71.0 (60.3, 81.0)	< 0.001

IQR, interquartile range; AMI, acute myocardial infarction; UAP, unstable angina pectoris.

### Association between weather conditions and AMI hospitalizations

**Figure 1:**
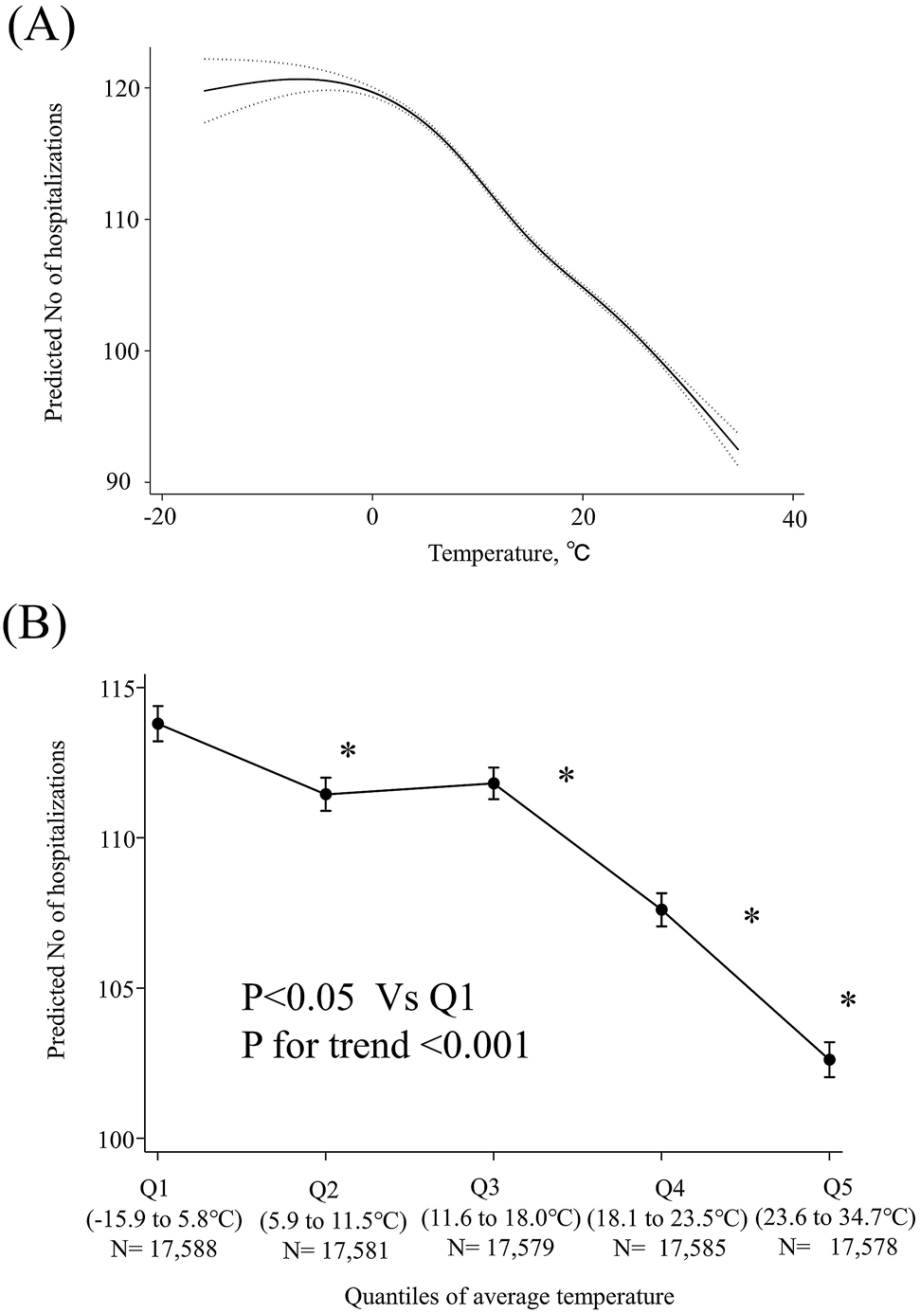
Association between average weather temperature and acute myocardial infarction hospitalizations. (**A**) MVRS (multivariable regression splines) indicated a linear relationship with temeprature. The predicted number of AMI per day was univariate. The x-axis represents temperature (°C) as a continuous variable. The solid and dashed lines indicate 95% confidence intervals. (**B**) The adjusted predicted number of AMI per day was calculated using “marginsplot” after the creation of multilevel mixed random-effects and population-averaged linear models in STATA. The covariates are the same as in Table 2. The x-axis represents temperature (°C) as a categorical variable according to the weather temperature quantiles. Bars indicate 95% confidence intervals. Q, quantile. The association of hospitalization with temperature was close to linear.

The multilevel mixed-effects linear regression analysis shown in Table 2 indicates that a number of incident AMI hospitalizations were associated with lower average weather temperatures and humidity (coefficient − 0.500 [− 0.528 to − 0.474] per ℃, *p* < 0.001 and coefficient − 0.012 [− 0.023 to − 0.001] per %, *p* = 0.039, respectively) after adjustments for season, hospital, and patient demographics. Association of cumulative number of AMI hospitalization each month with temperature, and with humidity was shown in Supplementry Fig. 1.

**Table 2:** Association of number of AMI hospitalizations with temperature and humidity.

	Multilevel mixed-effects liner regression Random effect; Institution	
	Adjusted coefficient (95% CI)	P value
Average temperature, °C	− 0.500 (− 0.528 to − 0.474)	< 0.001
Average humidity, %	− 0.012 (− 0.023 to − 0.001)	0.039

Coefficients represent the change in number of AMI hospitalizations per 1 unit increase in temperature or humidity with adjustments for multiple variables: season, number of hospital beds, coronary care units, cardiac surgery, east–west Japan, age, gender, height, weight, brinkman index, Charlson score, and average humidity or average temperature.

### Modelling temperature and humidity effects as flexible curves

Figure 1A indicates that there was a negative association between average temperature and the adjusted number of AMI hospitalizations. Average temperatures were divided into five quantiles, indicating a decreased adjusted number of AMI hospitalizations with increasing average temperature (Fig. 1B, p for trend < 0.001).

**Figure 2:**
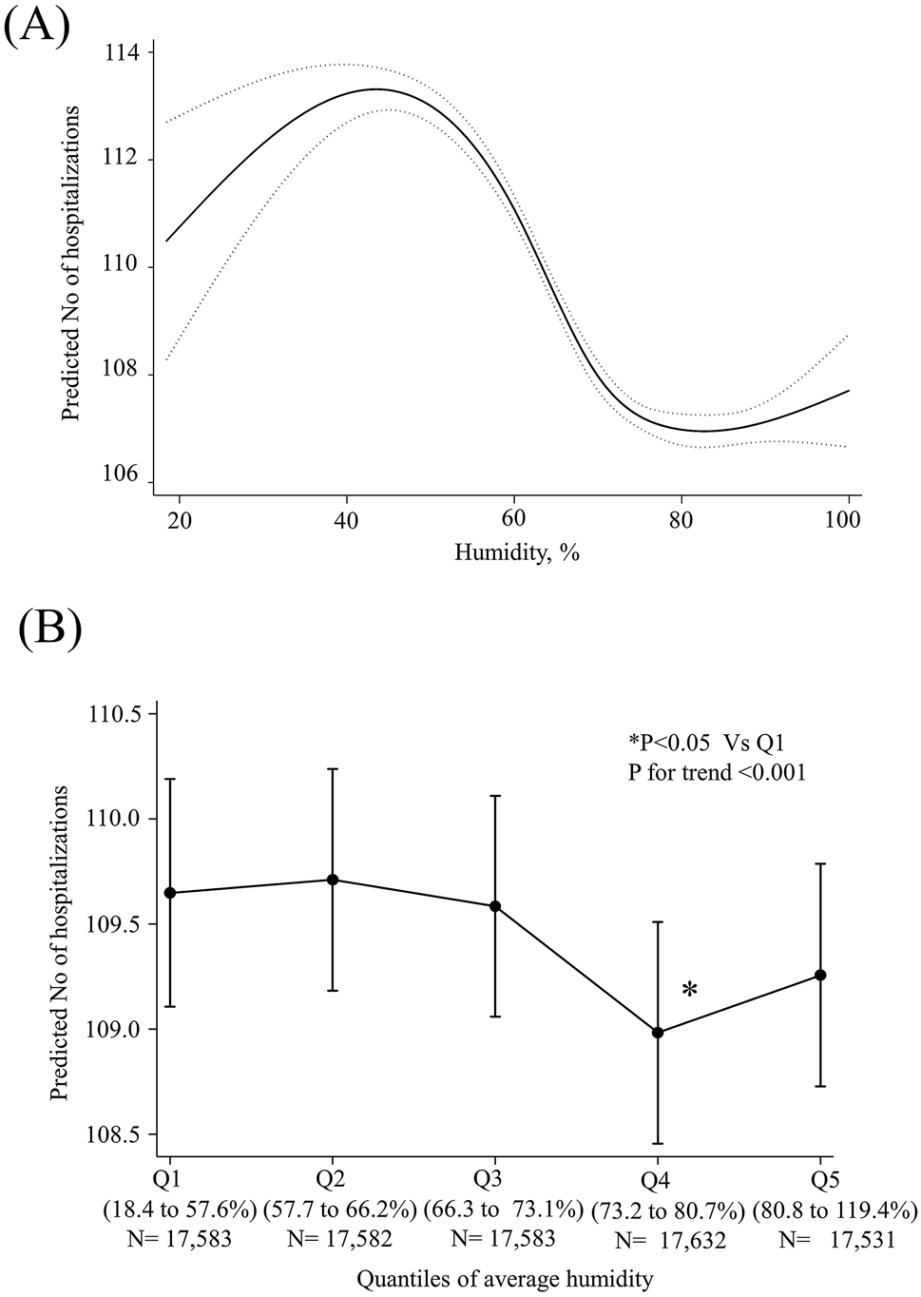
Association between average weather humidity and acute myocardial infarction hospitalizations. (**A**) Linearity was checked for continuous and categorical variables using STATA's multivariable regression splines (MVRS) command. MVRS indicated non-linear relationship with humidity. The predicted number of AMI per day was univariate. The x-axis represents humidity (%) as continuous variables. The solid and dashed lines indicate 95% confidence intervals. (**B**) The adjusted predicted number of AMI per day was calculated using “marginsplot” after the creation of multilevel mixed random-effects and population-averaged linear models in STATA. The covariates are the same as in Table 2. The x-axis represents humidity (%) as categorical variables according to weather humidity quantiles. The bars indicate the 95% confidence intervals. Q, quantile. The association between hospitalizations and weather humidify was close to non-linear; Q4 had the lowest risk.

There was a non-linear relationship between humidity and the number of AMI hospitalizations (Fig. 2A). A positive linear association was found between average temperature and AMI hospitalization at an average humidity of < 50% (coefficient 0.091 [0.029 to 0.152] per %, *p* = 0.004) in Table 3. However, a negative linear association was found at an average humidity of 50–80% (coefficient − 0.037 [− 0.055 to − 0.019] per %, *p* < 0.001). When classified into five quantiles of average humidity, the number of AMI hospitalizations in Quantile 1 was significantly lower than that in Quantile 4 (Fig. 2B, p < 0.05). This indicates that Quantile 4 of humidity posed the lowest risk.

**Table 3:** Association of number of AMI hospitalizations with humidity for subgroup.

	Multilevel mixed-effects liner regression Random effect; Institution	
	Adjusted coefficient (95% CI)	P value
Average humidity < 50%	0.091 (0.029 to 0.152)	0.004
Average humidity 50–80%	− 0.037 (− 0.055 to − 0.019)	< 0.001
Average humidity > 80%	0.034 (− 0.009 to 0.078)	0.125

Coefficients represent the change in number of AMI hospitalizations per 1 unit increase in humidity with adjustments for multiple variables: average temperature, season, number of hospital beds, coronary care units, cardiac surgery, east–west Japan, age, gender, height, weight, brinkman index, Charlson score, and average temperature.

### Effects of weather condition indices in subgroups

**Figure 3:**
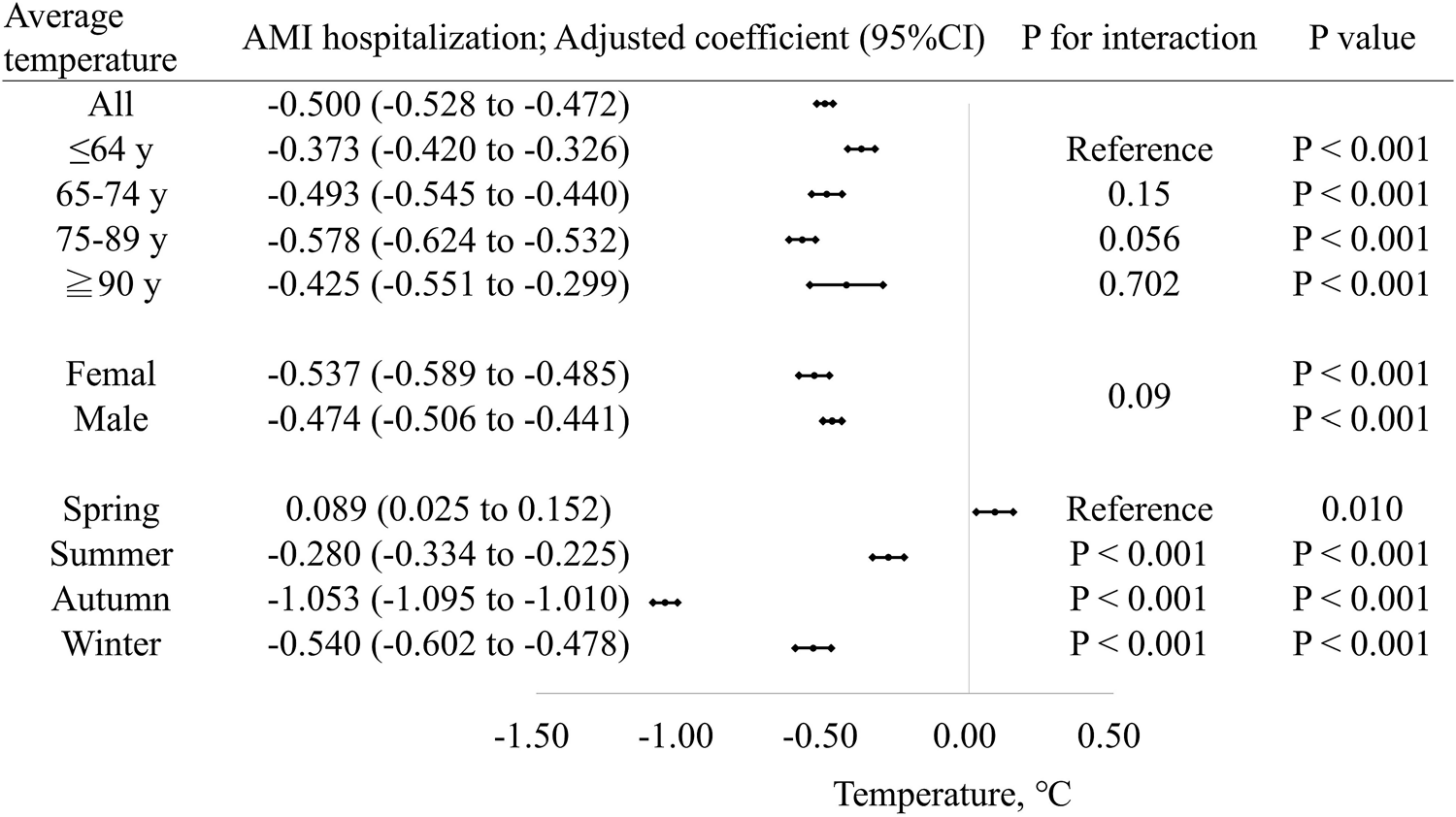
Association between average weather temperature and acute myocardial infarction hospitalizations for several subgroups. Coefficients greater than zero represent an increase in the number of cardiovascular hospitalizations by the average weather temperature. The coefficient is indicated by a dot, and the lines represent the 95% confidence intervals. Multilevel mixed random-effects and population-averaged linear models was used and the coefficients were adjusted as indicated in Table 2. Lower temperatures increase the risk of AMI hospitalization, except in spring.

The association between average weather temperature and specific risk estimates of incident AMI is summarized in Fig. 3. The effects of average weather temperature on AMI admissions tended to be greater in the 75–89 years age group than in the ≤ 64 years age group (coefficient − 0.578 [− 0.624 to − 0.532] vs. ≤ 64 years age group, interaction *p* = 0.056). However, there was no difference between the ≤ 64 years(interaction *p* = 0.150) and the ≥ 90 years (interaction *p* = 0.702) age groups. The effects of average weather temperature did not differ between male and female patients (interaction *p* = 0.090). Among the four seasons, the effect of average temperature on AMI admission was most pronounced in autumn (coefficient − 1.053 [− 1.095 to − 1.010] vs. spring, interaction *p* < 0.001) followed by winter (coefficient − 0.540 [− 0.602 to − 0.478] vs. spring, interaction *p* < 0.001). In spring, however, the increased average weather temperature was associated with a greater number of AMI hospitalizations (coefficient 0.089 [0.025 to 0.152], interaction *p* = 0.01).

**Figure 4:**
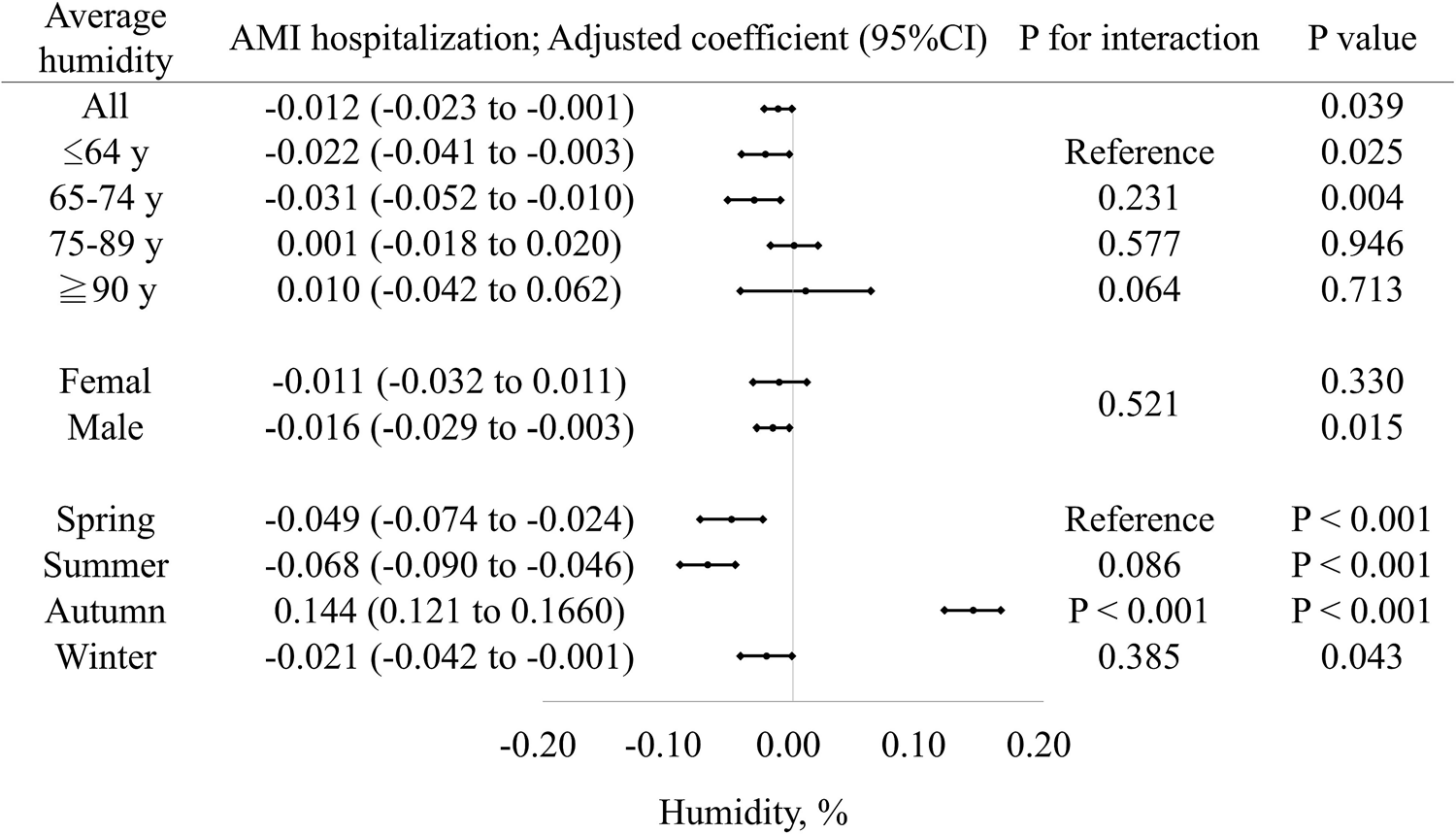
Association between average weather humidity and acute myocardial infarction hospitalizations for several subgroups. Coefficients greater than zero represent an increase in the number of cardiovascular hospitalizations by the average weather temperature. The coefficient is indicated by a dot, and the lines represent the 95% confidence intervals. Multilevel mixed random-effects and population-averaged linear models was used and the coefficients were adjusted as indicated in Table 2. Overall, lower humidity increases the risk of AMI hospitalization in autumn.

No difference in the effects of average humidity was found among age categories and between sexes (Fig. 4, all interaction p > 0.05). In contrast to other seasons, the increase in average humidity was associated with a greater number of AMI hospitalizations in autumn (coefficient 0.144 [0.121 to 0.166] per %, interaction *p* < 0.001).

## Discussion

Using a Japanese nationwide registry dataset of 87,911 patients with AMI, we evaluated the impact of climate conditions on AMI hospitalization in the super-aging society of Japan. The main findings were as follows: (1) lower temperatures were associated with an increased number of AMI hospitalizations: (2) lower humidity was associated with an increased number of AMI hospitalizations and Quantile 4 of humidity posed the lowest risk; (3) the effect of temperature and humidity on AMI hospitalization was consistent across age groups and sex, but responses varied by season. Overall, this study strongly suggests that protection against the cold and low humidity is important for the prevention of incident AMI in the super-aging society of Japan. To date, thera have been few reports clarifing the association between humidity and the incidence of AMI. A major strength of this study is the large number of subjects as compared to the previous studies^9,13,14^, and this nationalwide JROAD dataset covers various types of cardiovascular hospital in every region of Japan.

### Low temperature and high AMI hospitalization

Several epidemiological studies have reported that lower temperatures are a risk factor for the incidence of AMI^4,15–17^. These studies support our results. Compared to previous studies, we found that the effect of temperature on AMI hospitalization was consistent across age groups and sex in the super-ageing society of Japan. The mechanisms underlying the cold-induced increased risk of incident AMI are not fully understood. Cold temperatures lead to a rise in catecholamine levels^18^, resulting in increased heart rate and blood pressure, and subsequent myocardial ischemia and plaque vulnerabilities. Cold temperatures also increase serum cholesterol^19^, platelets, and fibrinogen activity, leading to elevated blood viscosity^20^. In particular, rapid exposure to low temperatures is known to cause coronary spasm^21^.

Contrary to our expectations, we found that the response to AMI risk factors was consistent across age groups and sexes. Other studies from Hong Kong and Taiwan reported that the association of low temperature with AMI risk was stronger for the 65–74 and > 75 years age groups compared with the ≤ 64 age group^22^. Similarly, our data shows that the 75–89 years age group was most affected by the low temperatures. Cold-induced changes in blood pressure^23^, plasma cholesterol, and fibrinogen^20^ have been reported to be more pronounced in the elderly. This may result in a higher risk of incident AMI with a decrease in temperature in the elderly than in younger people. In contrast, our study found that the effect of temperature on people aged over 90 years did not differ from that of younger people. This may have been due to the higher rate of medication intake and less outdoor activity in the ≥ 90 years age group. Alternatively, we considered the possibility that the effect of “age” was greater than the effect of temperature, thereby masking the association with AMI risk.

### Low humidity and high AMI hospitalization

We found that lower humidity was associated with an increased number of AMI hospitalizations. Lee et al. used the Korean AMI registry (n = 2136) to investigate the association between meteorological parameters and hospital admission for AMI^9^. They found a negative correlation between hospital admission for AMI and relative humidity, which supports our results. In contrast, Panagiotakos et al., in a study of admissions for acute coronary syndromes in the Athens area (n = 5458), found relative humidity to be positively correlated with hospital admission^12^. Swartz et al. estimated the effects of humidity on hospital admissions for heart disease and myocardial infarction in individuals aged > 65 years in 12 U.S. cities, and showed that there was no evidence of a humidity effect^11^. The inconsistent results may be due to differences in research locations and races, because studies^9,13,14^ conducted in East Asia, including the present study, showed a tendency toward a negative association.

Unlike most previous studies, however, our study demonstrated that humidity Q4 posed the lowest risk of hospitalization for AMI. Mohammad et al. conducted a prospective, large scale, population-based, nationwide observational study (n = 274,029) and reported a U-shaped association between humidity and AMI incidence. In their report, the lowest risk of AMI was at 70–80% humidity, which supports our results. Therefore, it may be important to avoid extremely high and low humidity.

The exact mechanism of the increased incidence of AMI at very high and low humidity still remains unknown. It is hypothesized that very high humidity may interfere with the processes of perspiration and body temperature homeostasis, resulting in respiratory fatigue and increased heart rate. On the other hand, very low humidity and low temperatures can interact with each other to increase the incidence of respiratory diseases and influenza, which in turn can increase AMI incidences.

### The effect of temperature or humidity on AMI hospitalization varied by season

We found that the most pronounced effects of lower and higher temperatures were observed in autumn and spring, respectively. A study in Belgium showed pronounced effects of temperature decreases in summer; however, the effects were not detected in winter^4^. A SWEDEHEART nationwide observational study reported a negative correlation between minimum temperatures and the incidence of AMI from April to December but not from January to March^10^. Yamaji et al., using a nationwide database, showed that an increased mean temperature was independently associated with lower AMI hospitalization numbers in winter but not in summer^13^. In summary, except for the study by Yamaji et al.^13^, the effects of low temperatures on increased AMI admissions have been reported to be greater in seasons other than winter. The reason behind higher temperatures in spring increasing AMI hospitalization is unknown. In spring, the sudden rise in temperatures from the cold winter may be related to the onset of AMI. The magnitude of the effect of the temperature change may be as equally important as the absolute value of the temperature.

Additionally, higher humidity in the autumn is associated with AMI onset, but there is no data to explain the differences in seasonal responses. Potential mechanisms include seasonal differences in blood pressure reactivity^24^, heart rate variability^25^, weight change^26^, cholesterol levels^19^, and endothelial dysdunction^27^. Further studies are needed to confirm the effects of seasonal variations in temperature and humidity.

### Fighting against cold temperature and low humidity

Hospitalization for AMI is a growing public health concern in the super-aging society of Japan. Therefore, the results of the present study have important implications for reducing the risk of AMI. The findings may guide public health interventions to control and prevent the cardiovascular effects of exposure to cold temperatures and low humidity, particularly for the very elderly who are at a high risk of hospitalization due to coronary artery disease. In addition to keeping warm, keeping humid is also important, especially during winter. Maintaining the main rooms at the right temperatures and humidity can prevent factors that trigger incident AMI.

### Limitations

There are several limitations to this study that should be acknowledged. First, our study included only Diagnosis Procedure Combination (DPC) hospitals with cardiovascular beds that meet the requirements of the Japanese Circulation Society (JCS). Although these institutions account for 29% of all hospital beds in Japan, the applicability of our findings to non-certified or non-specialist hospitals is unclear. However, the JROAD is the largest cross-sectional study of nationwide cardiac health outcomes in Japan and constitutes a comprehensive database of epidemiological data for population-based studies. Second, cross-sectional studies do not determine cause and effect; however, the large sample size is a noteworthy strength of this study. Future longitudinal studies of the mechanism of the temperature/humidity effects on AMI events and their outcomes are warranted. Determinants of the populations that are most vulnerable to weather changes should be further explored. Third, the present study could not include some important confounding factors such as medication before hospital admission and type of AMI (ST-segment elevation or non-ST-segment elevation). The diagnosis of atrial fibrillation was defined from the JROAD-DPC database and not from the electrocardiogram. Therefore, this study would underestimate the comorbidities. Finally, Japan is long in east–west and north–south directions. The regional differences such as Okinawa and Hokkaido were adjusted for east–west as covariate factors, but regional effects need to be examined in the future.

## Conclusions

Lower weather temperatures and humidity are both associated with an increased incidence of AMI hospitalizations in the aging society of Japan. The risk of AMI hospitalization did not differ with age or sex, but did differ by season. Higher temperatures in spring and higher humidity in autumn tended to increase the risk of AMI hospitalizations. The findings of this study can provide guidance to healthcare providers on advising patients to be watchful of weather temperature and humidity in a country with an increasingly aging society.

## Methods

### Data collection

The JROAD database is a nationwide prospective registry. The database was designed to assess the clinical activity of each Japanese institution regarding cardiovascular care, and to provide adequate feedback to teaching hospitals for improving patient care. A detailed description of the database design and methods was previously published^28^. Briefly, the JCS developed the JROAD database, which includes the demographics of each hospital since 2004. The JCS also developed the JROAD-DPC nationwide database, which includes data from the Japanese diagnosis procedure combination/per diem payment system (DPC/PDPS) since 2014. The DPC database is a mixed-case classification system linked with a lump-sum payment system, which was launched in 2002 by the Japanese Ministry of Health, Labour and Welfare^29^. Compared with other registry databases, the Japanese DPC database enables researchers to conduct nationwide studies of both descriptive and/or analytical epidemiology in a real-world clinical setting. The DPC database includes data on the following elements: demographics for each patient (e.g., age and sex); principal diagnoses (coded according to the International Classification of Diseases, 10th revision [ICD-10]); comorbidities at admission (ICD-10 coded); complications after admission (ICD-10 coded); procedures, including surgery, medications, and devices used during hospitalization; length of stay; discharge status; and medical expenses^28–31^. Institutions using the DPC system encompass a wide variety of centers, including academic, urban, and rural hospitals^28,32^. All data included in this study were from hospitalized patients with clinically apparent cardiovascular disease. We collated and used a dataset of weather variables in Japan from the National Institute for Environmental Studies (http://www.nies.go.jp/db/index-e.html). The weather variables included hourly weather temperatures and humidity. Localized weather data were obtained by identifying the weather station closest to each hospital. We searched the sites of weather stations by the municipality code which the Ministry of Internal Affairs and Communications in Japan assigns to each municipality specifically and we merged the sites of the hospitals and the monitoring stations closest to them. The weather variables were merged with the DPC database using the acute-hospitalization day and the municipal code provided by the Japanese Ministry of Internal Affairs and Communications (http://www.soumu.go.jp/denshijiti/code.html).

**Figure 5:**
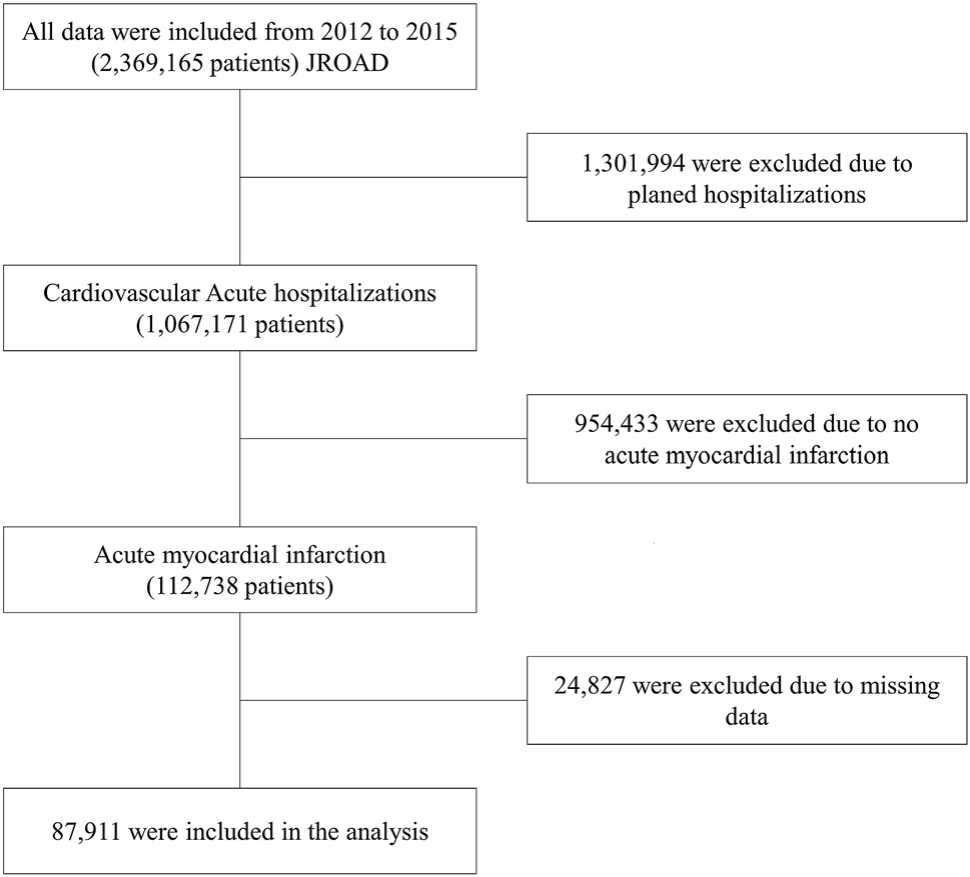
Flowchart of the present study. JROAD, Japanese registry of all cardiac and vascular diseases.

This study included 911 hospitals, where 2,369,165 consecutive patients were admitted during the study period. We collected the data of patients with AMI requiring hospitalization among these data. The exclusion criteria were as follows: planned hospitalization; no AMI; missing temperature or humidity data; and missing patient-characteristic data. Finally, 87,911 patients were included in the analysis (Fig. 5).

This study was conducted according to the principles of the Declaration of Helsinki. The present research was designed by the authors, and the study protocol was approved by the institutional ethics committee of St. Marianna University of Medicine. Each hospital anonymized patient IDs using code-change equations for the original JROAD-DPC data. The requirement for individual informed consent was waived by the institutional ethics committee of St. Marianna University of Medicine because all of the data were basically anonymized when provided by the DPC, which was sent to the Ministry of Health, Labour and Welfare, and National Cerebral and Cardiovascular Center managed the database. National Cerebral and Cardiovascular Center notified patients that their information was being collected by this study through homepages or posters in each hospital. Patients could choose to have their information excluded.

This study did not investigate data on blood tests or electrocardiography because we used only DPC data. AMI is assigned International Statistical Classification of Diseases and Related Health Problems 10th Revision **(**ICD-10) code I21, which is recorded in the “main diagnosis,” “admission precipitating diagnosis,” “most resource-consuming diagnosis,” or “second most resource-consuming diagnosis.”

### Exposure

Seasons were separated into spring (March–May), summer (June–August), autumn (September–November), and winter (December–February). The average temporaries were defined as the average of each hourly temperature and humidity within a day. The averages of weather variables on a certain day were assigned to the day before an emergency hospitalization due to AMI. This is because hospitalization times were not available in the database.

### Design

We conducted a cross-sectional study using data from the JROAD and JROAD-DPC, and weather variables between April 1, 2012 and March 31, 2015.

### Outcomes

The primary outcome was the number of AMI cases requiring hospitalization per day.

### Covariates

The average weather temperature and humidity, as continuous variables, were adjusted for season, hospital demographics (east/west Japan, number of hospital beds, and the presence of a coronary care unit, cardiac surgery service, and a board-certified cardiologist), and patient demographics (age, sex, height, weight, smoking, and Charlson comorbidity index).

### Statistical analysis

Patient characteristics are expressed as medians and interquartile ranges for continuous variables. Categorical variables are presented as frequencies (%). We used multilevel mixed random effects and population-averaged linear models to evaluate the association between the number of AMI hospitalizations and weather variables. The multilevel mixed-effect models were used to evaluate the random effects of hospital variations (institutional code) determined by the JROAD study. The covariates were considered clinically important factors by experienced cardiologists. The average weather temperature and humidity, as continuous variables, were adjusted for the covariates. Linearity was checked for continuous and categorical variables using STATA's multivariable regression splines (MVRS) command and the 5th percentile, respectively. MVRS selects the regression spline model that best predicts the outcome variable. MVRS indicated a linear spline relationship with humidity. The cuff-off value of humidity was determined based on the knots calculated by MVRS and was tested using “mkspline” command in STATA. The mkspline creates variables containing a linear spline. For subgroup analysis, we classified the patients’ age into 4 groups according to the Japan Geriatrics Society guidelines^33^. The interaction was examined for age, gender, and season. All analyses were carried out using STATA statistical software, version 14 (Stata Corp., College Station, TX, USA). Statistical significance was defined as *p* < 0.05.

## Supplementary Information

Supplementary Figure 1.

## Data Availability

The data that support the findings of this study are available from the JROAD; however, restrictions apply to the availability of these data, which were used under approval for the current study and are thus not publicly available. Data are however available from the JROAD upon reasonable request. Environmental data are available from the National Institute for Environmental Studies, Japan (http://www.nies.go.jp/db/index-e.html).
