# Supplementary material for: Effects of temperature and humidity on acute myocardial infarction hospitalization in a super-aging society
Source: Sci Rep. 2021 Nov 24;11:22832. doi: 10.1038/s41598-021-02369-x (PMC8613245; doi:10.1038/s41598-021-02369-x)
Supplement: Supplementary file 1 — Supplementary Figure 1. [file 41598_2021_2369_MOESM1_ESM.pdf]

SUPPLEMENTAL MATERIAL

Effects of Temperature and Humidity on Acute Myocardial Infarction Hospitalization in a Super-aging Society

Takumi Higuma, Kihei Yoneyama,, Michikazu Nakai, Toshiki Kaihara, Yoko Sumita, Mika Watanabe, Shunichi Doi, Yoshihiro Miyamoto, Satoshi Yasuda, Yuki Ishibashi, Masaki Izumo, Yasuhiro Tanabe, Tomoo Harada, Hisao Ogawa, Yoshihiro J. Akashi

Supplemental Figure 1.

Supplemental Figure 1. Association of cumulative number of acute myocardial infarction hospitalization each month with temperature, and with humidity.

The bar represents cumulative number of acute myocardial infarction (AMI) hospitalization each month in Japan (April 2012-March 2015). The dashed line represents median of average daily humidity one day before AMI hospitalization. The solid line represents median of average daily temperature one day before AMI hospitalization.

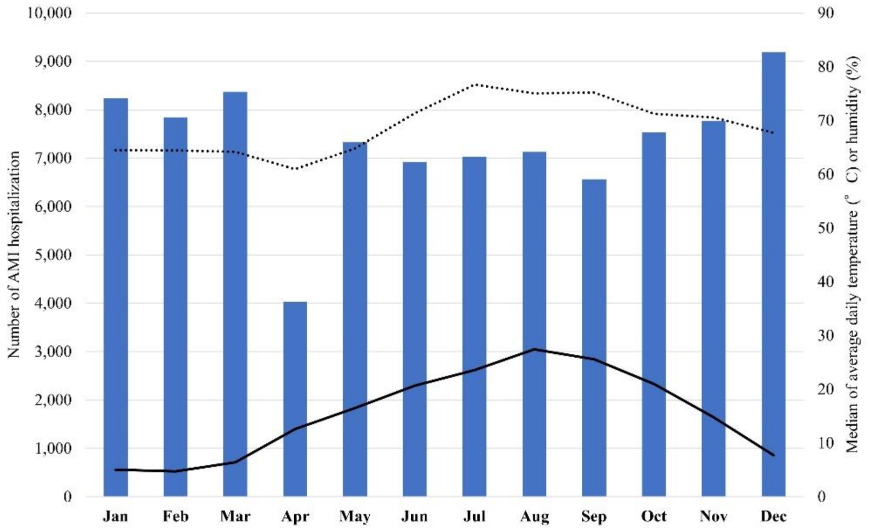

Supplemental Figure 1.
